# Tumor-to-bone distance and radiomic features on MRI distinguish intramuscular lipomas from well-differentiated liposarcomas

**DOI:** 10.1186/s13018-023-03718-4

**Published:** 2023-03-28

**Authors:** Narumol Sudjai, Palanan Siriwanarangsun, Nittaya Lektrakul, Pairash Saiviroonporn, Sorranart Maungsomboon, Rapin Phimolsarnti, Apichat Asavamongkolkul, Chandhanarat Chandhanayingyong

**Affiliations:** 1 Department of Orthopaedic Surgery, Faculty of Medicine Siriraj Hospital, Mahidol University, 2 Wanglang Road, Bangkoknoi, Bangkok, 10700 Thailand; 2 Department of Radiology, Faculty of Medicine Siriraj Hospital, Mahidol University, Bangkok, 10700 Thailand; 3 Department of Pathology, Faculty of Medicine Siriraj Hospital, Mahidol University, Bangkok, 10700 Thailand

**Keywords:** Artificial intelligence, Machine learning, Lipoma, Liposarcoma, Magnetic resonance imaging, Radiomics

## Abstract

**Background:**

To develop a machine learning model based on tumor-to-bone distance and radiomic features derived from preoperative MRI images to distinguish intramuscular (IM) lipomas and atypical lipomatous tumors/well-differentiated liposarcomas (ALTs/WDLSs) and compared with radiologists.

**Methods:**

The study included patients with IM lipomas and ALTs/WDLSs diagnosed between 2010 and 2022, and with MRI scans (sequence/field strength: T1-weighted (T1W) imaging at 1.5 or 3.0 Tesla MRI). Manual segmentation of tumors based on the three-dimensional T1W images was performed by two observers to appraise the intra- and interobserver variability. After radiomic features and tumor-to-bone distance were extracted, it was used to train a machine learning model to distinguish IM lipomas and ALTs/WDLSs. Both feature selection and classification steps were performed using Least Absolute Shrinkage and Selection Operator logistic regression. The performance of the classification model was assessed using a tenfold cross-validation strategy and subsequently evaluated using the receiver operating characteristic curve (ROC) analysis. The classification agreement of two experienced musculoskeletal (MSK) radiologists was assessed using the kappa statistics. The diagnosis accuracy of each radiologist was evaluated using the final pathological results as the gold standard. Additionally, we compared the performance of the model and two radiologists in terms of the area under the receiver operator characteristic curves (AUCs) using the Delong’s test.

**Results:**

There were 68 tumors (38 IM lipomas and 30 ALTs/WDLSs). The AUC of the machine learning model was 0.88 [95% CI 0.72–1] (sensitivity, 91.6%; specificity, 85.7%; and accuracy, 89.0%). For Radiologist 1, the AUC was 0.94 [95% CI 0.87–1] (sensitivity, 97.4%; specificity, 90.9%; and accuracy, 95.0%), and as to Radiologist 2, the AUC was 0.91 [95% CI 0.83–0.99] (sensitivity, 100%; specificity, 81.8%; and accuracy, 93.3%). The classification agreement of the radiologists was 0.89 of kappa value (95% CI 0.76–1). Although the AUC of the model was lower than of two experienced MSK radiologists, there was no statistically significant difference between the model and two radiologists (all *P* > 0.05).

**Conclusions:**

The novel machine learning model based on tumor-to-bone distance and radiomic features is a noninvasive procedure that has the potential for distinguishing IM lipomas from ALTs/WDLSs. The predictive features that suggested malignancy were size, shape, depth, texture, histogram, and tumor-to-bone distance.

**Supplementary Information:**

The online version contains supplementary material available at 10.1186/s13018-023-03718-4.

## Introduction

Lipomatous soft tissue tumors are a group of tumors that exhibit a variety of clinical behaviors. Lipomas are the most common soft tissue tumor, accounting for one-third of soft tissue tumors [1, 2]. Lipomas are benign adipocytic tumors, and they can be treated conservatively with careful observation. Surgical excision is necessary when a patient is symptomatic [3]. Local recurrence may occur if the surgical margin is not clear, but the chance is very low. However, intramuscular (IM) lipomas that are larger than 5 cm, deep-seated, and symptomatic can sometimes be difficult to distinguish from atypical lipomatous tumors or well-differentiated liposarcomas (ALTs/WDLSs). The World Health Organization uses the terms ALT and WDLS to represent tumors with identical histology but different anatomical locations and clinical outcomes [4, 5]. ALTs characterize extremity or upper trunk lesions, and WDLSs represent retroperitoneal or mediastinal lesions. ALTs/WDLSs are low-grade, malignant, adipocytic tumors that recur locally or dedifferentiate to high-grade sarcomas, but they rarely metastasize [6, 7]. The gold standard for the diagnosis of ALTs/WDLSs is histopathological evidence of lipoblasts and lipocytes, with immunohistopathological staining positive for murine double minute 2 (MDM2) or cyclin-dependent kinase 4 (CDK4) [8, 9]. Establishing a diagnosis before surgery is crucial to dictate the urgency of surgery timing, to have a well-planned incision (a larger wound for ALTs/WDLSs), and to manage surgical margins (intracapsular incisions for lipomas, and capsular removals for ALTs/WDLSs). However, computed tomography-guided biopsies and incisional biopsies often cannot identify the malignant cell foci from the small core of a tissue sample [10]. Frozen sections of tissue are also considered unsuitable because fatty tissue is often too friable and cannot be fixed.

Magnetic resonance imaging (MRI) is the most useful diagnostic tool for lipomatous soft tissue tumors, and it can distinguish IM lipomas from ALTs/WDLSs. MR images of IM lipomas show a uniform structure of adipose tissue with high intensity in both T1- and T2-weighted images, and low signal intensity with fat suppression. The MRI features of ALTs/WDLSs are similar to those of lipomas. However, features that suggest malignancy are (1) masses that are deep to the fascia; (2) masses that are larger than 10 cm; (3) the presence of thick septa (> 2 mm); (4) an enhancement on the post-contrast sequence; (5) a nodular or non-adipose mass-like area, and (6) a decreased percentage of fat [11–13]. In contrast, high-grade liposarcomas (including myxoid/round cell, pleomorphic, and dedifferentiated liposarcomas) show low signal intensity in T1-weighted images and heterogeneous high signal intensity in T2-weighted images. However, the interobserver reliability for diagnosis of IM lipomas and ALTs/WDLSs showed slight to the substantial agreement, based on Cohen’s kappa coefficient [14]. To reduce diagnostic uncertainty in medical image classifications using MRI and to prevent inadequate or excessive treatments, diagnoses must be made by an experienced musculoskeletal (MSK) radiologist.

Currently, interest in artificial intelligence is strong and is growing rapidly. To remedy the image classification problem, several methods have been proposed, such as machine learning and deep learning [15–23]. Recently, Tang et al. [20] reported that performing machine learning in distinguishing between all types of lipomas (subcutaneous and IM lipomas) and ALTs on preoperative MRI has shown greater precision than MSK radiologists. However, no studies have yet focused on IM lipomas and ALTs/WDLSs.

Consequently, the primary objective of this study was to develop a machine learning model based on tumor-to-bone distance and radiomic features derived from preoperative MRI images to distinguish IM lipomas from ALTs/WDLSs and compared with radiologists. Regarding feature importance insights in the ultimate model, we evaluated the radiomic features that have significant in differentiating IM lipomas from ALTs/WDLSs. Along with, we examined whether a decrease in the tumor-to-bone distance could be used to pathologically differentiate ALTs/WDLSs from IM lipomas and whether it affected the clinical behavior of WDLS.

## Materials and methods

### Patients

This retrospective study was carried out after receiving a certificate of approval from the ethics committee of our institute, with a waiver of informed consent. Data sets were obtained from 68 patients with IM lipomas or ALTs/WDLSs. All had undergone an MRI examination and a total excision, with the diagnosis confirmed by histopathology and immunohistochemistry or fluorescence in situ hybridization (FISH) examination (The tumor with MDM2 and CDK4 amplification revealed by immunohistochemistry/FISH was diagnosed as ALT/WDLS finally. On the other hand, the tumor that had no MDM2 and CDK4 amplification was defined as IM lipoma.), at our institution between 2010 and 2022. The final pathological results were used as the gold standard for the classification process. The inclusion criteria were defined as follows: (1) the final pathological diagnosis confirmed as IM lipoma or ALT/WDLS; (2) received total excision of primary tumors; and (3) received preoperative MRI protocol that including T1-weighted image (T1WI) sequence. The exclusion criteria were listed as follows: (1) the patients received anticancer treatments before MRI scan; (2) having a history of other cancers; and (3) poor quality image. According to these criteria, 38 patients with IM lipomas and 30 patients with ALTs/WDLSs were included in this study.

### MRI acquisition

The preoperative MRI of the patients was obtained using a variety of scanners and sequences. In this study, the T1WI sequence was the most available since it was part of the routine clinical protocol of our institution. Thus, we focused on this sequence. The MRI data sets comprised the axial, coronal, and sagittal planes of T1WI, all of which reveal soft tissue tumors most clearly. All images were acquired by 1.5 or 3.0 Tesla MRI scanners (Philips Healthcare; GE Medical System; Magnetom Siemens Healthineers). The MRI slice thickness was 5 mm (median, range 3–8). The repetition time (TR) and echo time (TE) were 590 ms (median, range 425–2259) and 12 ms (median, range 7–25), respectively. The detailed parameters for T1WI sequence are listed in Additional file 1: Table S1.

### Three-dimensional (3D) tumor segmentation

**Fig. 1:**
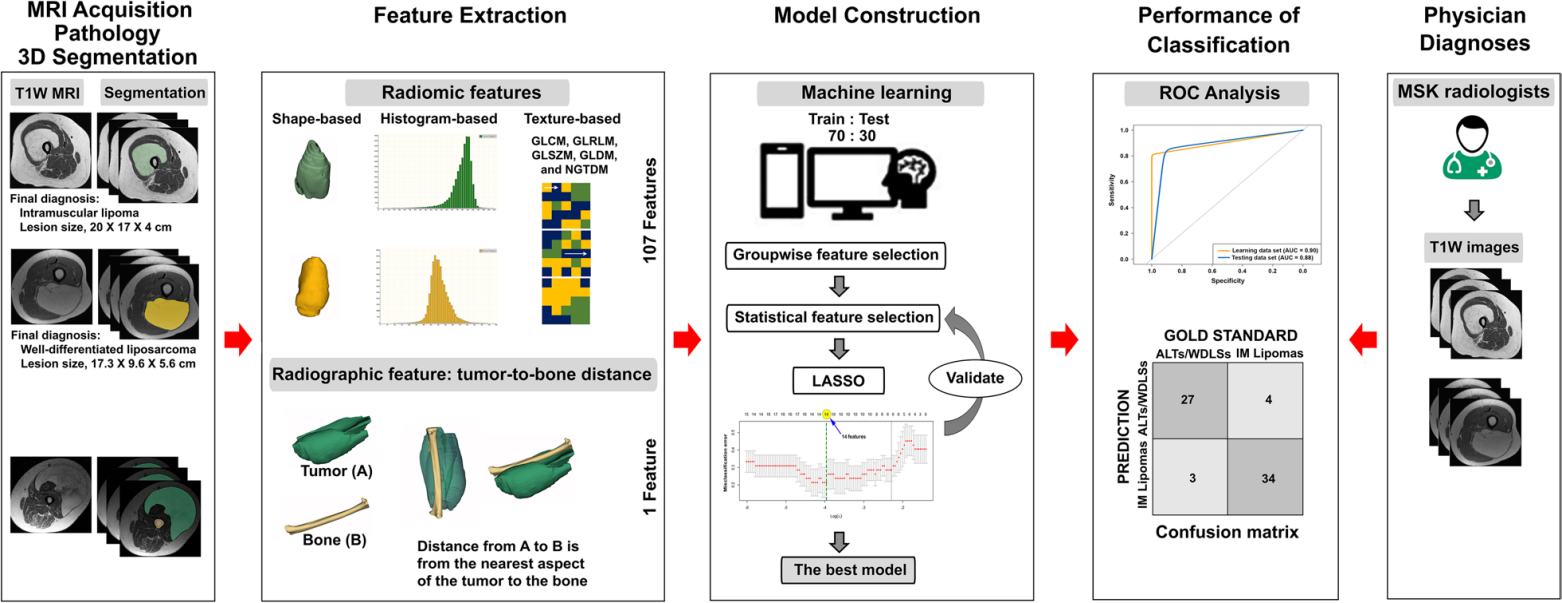
Study design diagram

The open-source software 3D Slicer version 4.11.20210226 r29738/7a593c8 (https://download.slicer.org/) with AI-assisted Nvidia Clara was used for manual segmentation of soft tissue tumors (Fig. 1). In all cases, lesions were segmented using the original T1WI sequences. Tumor region of interests (ROIs) were drawn on the entire volume of the lesion. Additionally, a reference region of interest (ROI) was drawn in fat on T1W MRI for image-intensity-normalization procedure [24]. All cases were selected randomly and blindly for repetitive the segmented ROI by two observers (statistician and research scientist) and subsequently confirmed their precision by experts in musculoskeletal radiology and orthopedic oncology. (One observer repeated the segmentation of all cases after a paused of 2 weeks to evaluate intraobserver variability. A second observer analyzed all cases to appraise interobserver variability.)

### Image-intensity normalization

After segmentation, the next step was image-intensity normalization. The purpose of this step was to adjust for differences in T1W MRI protocols. The normalized intensity value (NIV) [24] was determined as follows: $\text{NIV}=\frac{\text{Original T1W intensity}}{\text{Mean intensity value within the reference ROI}}\times 1000$.

In all cases, T1W MRI was normalized before radiomic feature extraction.

### Feature extraction

1. Radiomic features (107 features)

The radiomic features of a segmented ROI were extracted from the normalized T1W intensity using PyRadiomics version 3.0.1. Total radiomic features included first-order features (18 features); gray-level co-occurrence matrix (GLCM, 24 features); gray-level run length matrix (GLRLM, 16 features); gray-level size zone matrix (GLSZM, 16 features); gray-level dependence matrix (GLDM, 14 features); neighboring gray-tone difference matrix (NGTDM, 5 features); and shape-based features (3D, 14 features). These were classified as histogram-based features (i.e., first-order features), texture-based features (i.e., GLCM, GLRLM, GLSZM, GLDM, and NGTDM features), and shape-based features (Fig. 1) [25].

2. Radiographic feature: tumor-to-bone distance (1 feature)

**Fig. 2:**
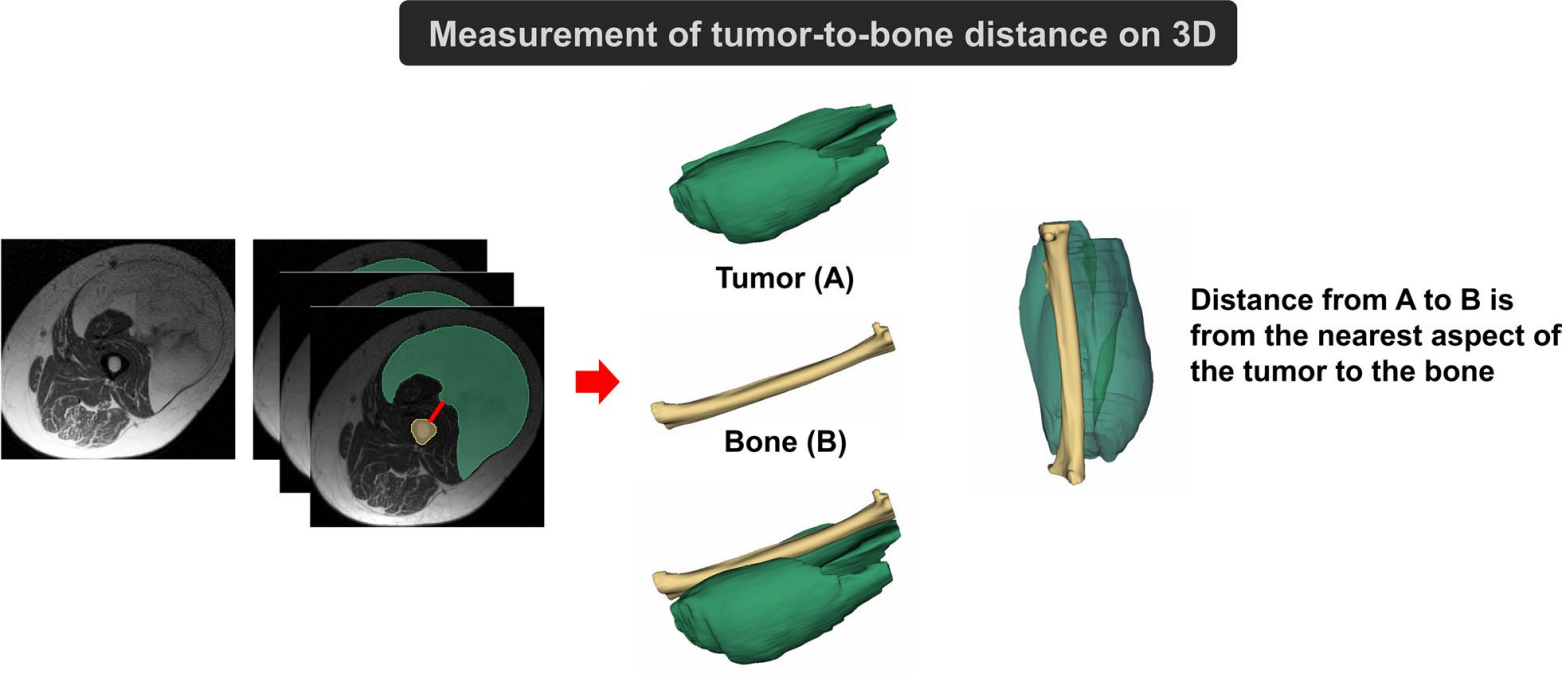
Measurement of tumor-to-bone distance on three-dimensional (3D) segmentation

The distance from each tumor to bone was measured in 3D (Figs. 1 and 2). The distance from A to B was from the closest aspect of the soft tissue tumor (A) to the bone (B).

### Classification by machine learning model

**Fig. 3:**
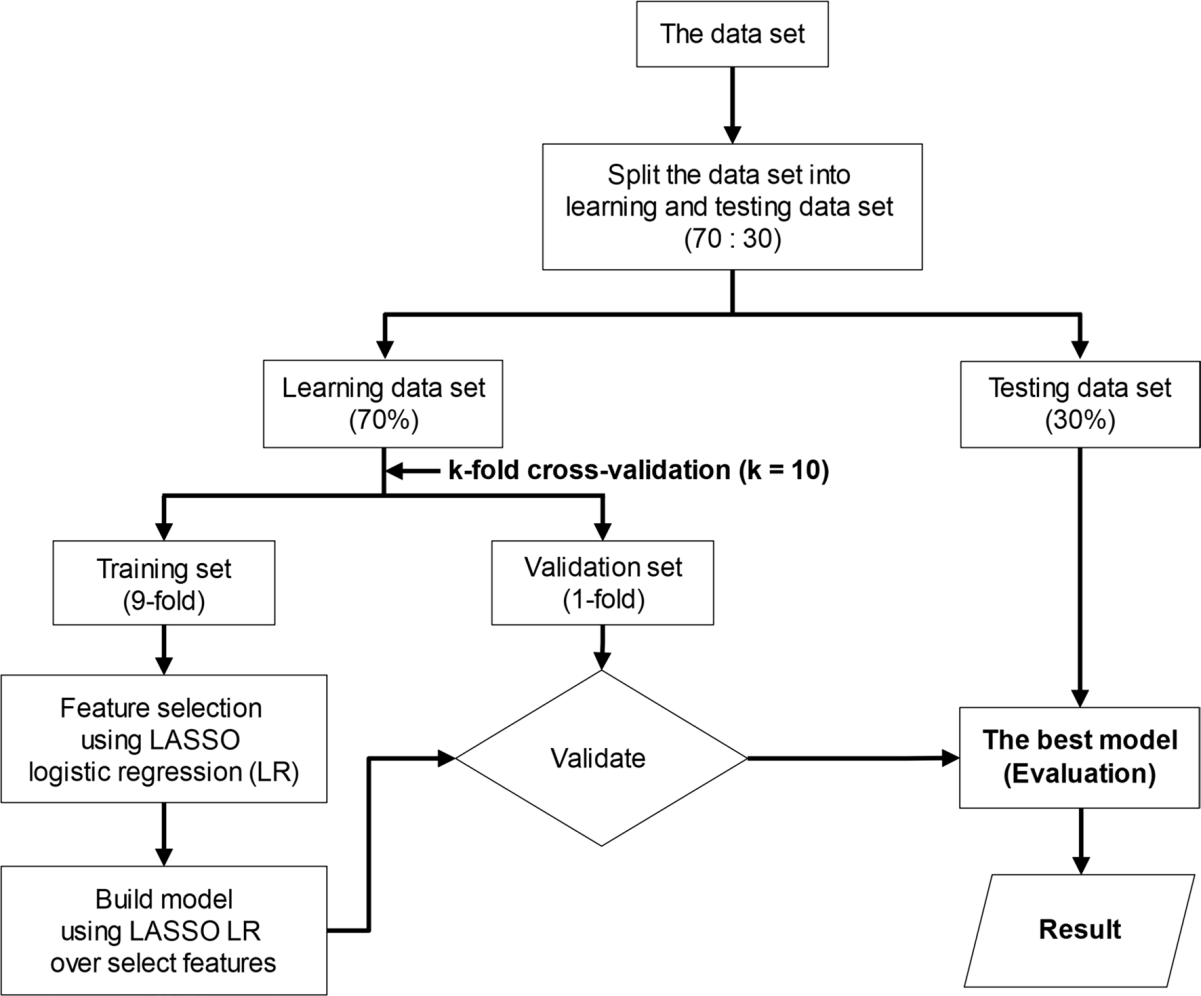
Workflow diagram of the machine learning process used to appraise the performance of classification models. The initial data set is split into two subsets: the learning and testing data sets. Afterward, the learning data set undergoes a tenfold cross-validation strategy when training sets are used to select features (“Feature selection” using LASSO logistic regression) and validation sets to test the performance of classification models. (For model building step, we apply LASSO logistic regression over selected features.) Finally, the best model is selected and evaluated on the testing data set (“Evaluation”)

**Fig. 4:**
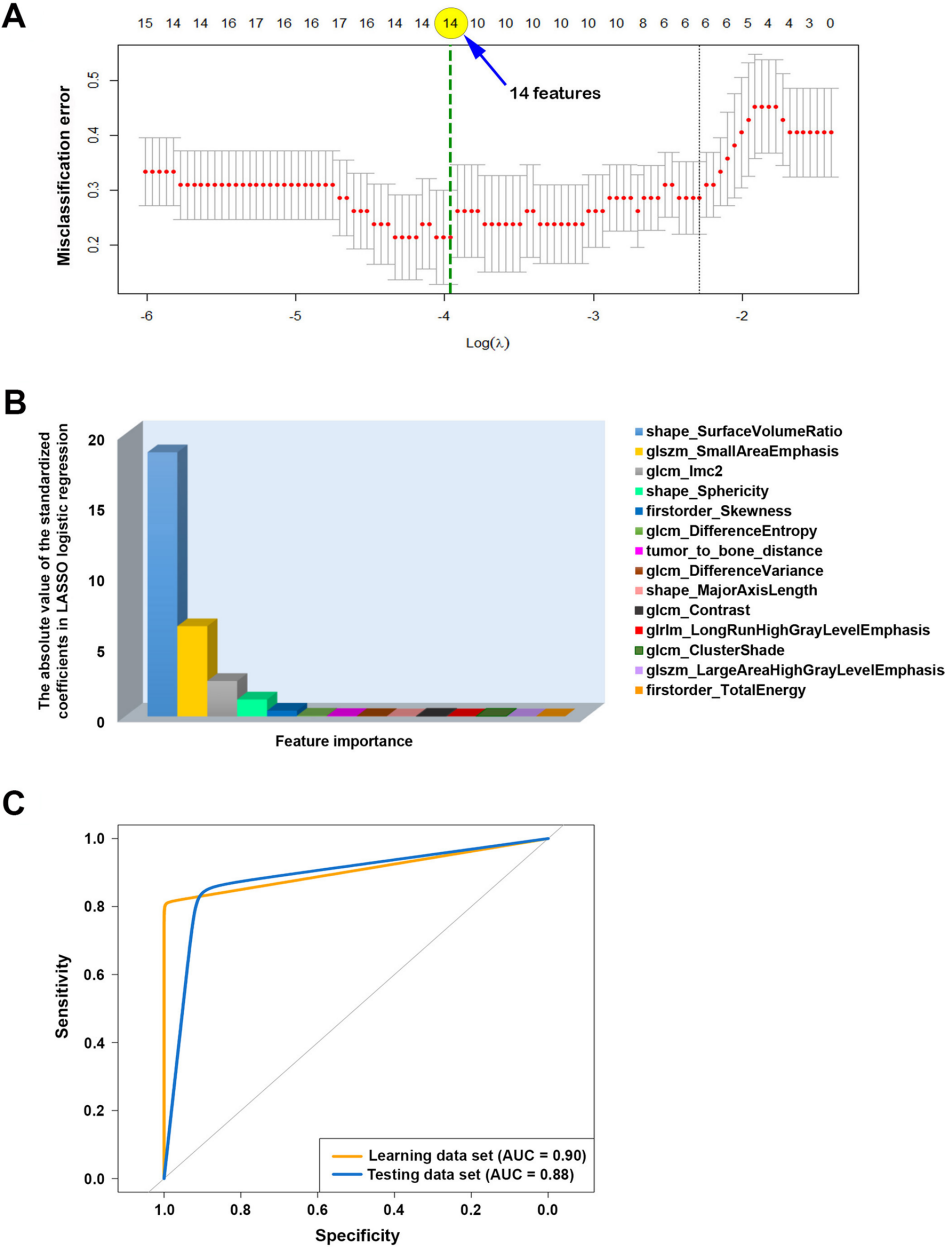
Fourteen features important in LASSO logistic regression model to distinguish IM lipomas from WDLSs: **A** selecting an optimal value of tuning parameter ($\lambda$) in the LASSO logistic regression model was conducted using tenfold cross-validation. The misclassification error was plotted against $\log (\lambda )$. $\lambda$ of 0.019 ($\log (\lambda )$ = − 3.96) was selected according to tenfold cross-validation. The green dash vertical line denotes the optimal value using minimum criteria; **B** fourteen features’ importance was obtained using the LASSO logistic regression model. The bar chart of the absolute standardized coefficients showed the feature importance ranking; and **C** receiver operating characteristic (ROC) curves were plotted for learning and testing data sets showing the area under the curves (AUCs) obtained using the LASSO logistic regression model

To predict soft tissue tumor differentiation, a classification model was constructed with supervised machine learning. Firstly, the data sets were randomly split into two subsets: the learning and testing data sets. (70% of data were used for the learning data set, and 30% for the testing data set.) Then, we applied a tenfold cross-validation strategy to the learning data set, which was repeatedly subdivided into training sets (ninefold) and validation sets (onefold). The training sets were used for “Feature selection” using LASSO logistic regression, and subsequently built models using LASSO logistic regression. This method is suitable for high-dimensional data [26]. For the validation sets were used to test the performance of the built model. Finally, the best model is selected and then evaluated on the testing data set (Fig. 3) [26, 27]. The performance of the classification model was assessed using the area under the curve (AUC), sensitivity, specificity, and accuracy (Fig. 4C) [27].

### Classification by radiologists

Two MSK radiologists served as readers, blindly and independently, to label the same lipomatous tumor MRI test set above. Radiologist 1 and 2 had 7 and 22 years of experience retrospectively. The readers were shown T1W MRI sequences in portable network graphics (PNG) format with an image size of 1024 × 1024 pixels; they were able to zoom in and out using the software. They made binary decisions on the 68 MR images and were blinded to the reports. The ratio of normal to abnormal radiographs was also not revealed to the readers. The classification agreement of the 2 radiologists was evaluated with kappa statistics [28].

### Statistical analysis

Mean and standard deviation (SD) or median and range were used to describe the continuous data, and frequency and percentage were used for categorical data. Comparison of the characteristics and the properties of the acquisition protocols of T1W MRI sequences between 2 groups (IM lipomas vs. ALTs/WDLSs) were analyzed using an independent sample t test or the Mann–Whitney U test for continuous data, and the chi-squared test or Fisher’s exact test for categorical data.

Regarding assessing intra- and interobserver variability of manual segmentation, the reproducibility of the radiomic features and tumor-to-bone distance extracted from normalized images were used to appraise the agreement of the feature values concerning intra- and interobserver variability by using the intraclass correlation coefficient (ICC). In the analysis, we chose a two-way random-effects model with an absolute agreement (ICC (2,1)) [29]. Interpretation of ICC value is as follows: poor (ICC < 0.50); moderate (0.50 ≤ ICC < 0.75); good (0.75 ≤ ICC < 0.90); and excellent (ICC ≥ 0.90) [30]. In this study, we considered a feature reproducible if the ICC value was more than 0.75 [24, 31–35]. Additionally, Pearson correlation coefficient (*r*)/Spearman’s rank-order coefficient ($\rho$) were also computed. The only features with ICC > 0.75 were utilized for machine learning procedure and statistical analysis.

Both the machine learning model and MSK radiologists, the area under the receiver operator characteristic curve (AUC), sensitivity, specificity, and accuracy values with 95% confidence interval (CI) were used to assess the performance of classification [27]. In addition, we compared the performance of the model and two radiologists in terms of the AUCs using the Delong’s test [36, 37].

Analysis of feature importance, differences in radiomic and radiographic features between two groups (IM lipomas vs. ALTs/WDLSs) were performed using the Bonferroni-corrected Mann–Whitney U test [22, 38, 39].

Statistical analysis and graphs were performed using R version 4.2.1 (R Foundation for Statistical Computing, Vienna, Austria). A* P* value < 0.05 was considered statistically significant.

## Results

### Patients

Sixty-eight patients were identified: 38 with IM lipomas and 30 with ALTs/WDLSs (Table 1). Most were women (61.8%) with deep-seated soft tissue tumors located in the thigh. The proportion of women in the IM lipoma group was significantly higher than in the ALT/WDLS group (*P* = 0.02). The mean age of the 68 patients was 59.3 years (range 38–80). There was no statistical difference in the mean ages of the IM lipoma and ALT/WDLS groups (*P* = 0.5).

**Table 1:** Demographic data of patients

Variable	Total subjects (*n* = 68)	IM lipomas (*n* = 38)	ALTs/WDLSs (*n* = 30)	*P* value
*Gender*				
Male	26 (38.2%)	10 (26.3%)	16 (53.3%)	0.02
Female	42 (61.8%)	28 (73.7%)	14 (46.7%)	
*Age (years)*				
Mean (SD)	59.3 (9.7)	60.0 (9.1)	58.1 (10.6)	0.5
Range	38–80	38–80	39–80	
*Tumor location*				
Thigh	34	14	20	
Arm	15	11	4	
Shoulder	9	9	0	
Leg	7	1	6	
Back/trunk	2	2	0	
Buttock	1	1	0	

*IM lipomas* intramuscular lipomas, *ALTs/WDLSs* atypical lipomatous tumors/well-differentiated liposarcomas

### Intra- and interobserver manual segmentation variability in the feature reproducibility

Intra- and interobserver variability of manual segmentation was appraised using ICC. The only features with ICC > 0.75 (78.7%, 85 of 108 features) were considered for feature selection and model building steps. Additionally, Pearson’s correlation coefficient and Spearman’s rank-order coefficient were more than 0.7 (Additional file 1: Table S2).

### The selected features in the ultimate model

The feature selection process with LASSO method revealed 14 significant features including 13 radiomic features (i.e., 3 shape-based features, 2 histogram-based features, and 8 texture-based features) and tumor-to-bone distance (Fig. 4A, B). The definition and formula of these features were described according to PyRadiomics’s documentation. (https://pyradiomics.readthedocs.io/en/latest/features.html) (Additional file 1: Table S3). All 14 were used to build the ultimate model in the next step.

### Performance of machine learning model

The classification performance of the machine learning approach used in this study is summarized in Table 2 and Fig. 4C, which performed well in differentiating IM lipomas from ALTs/WDLSs. The AUC, sensitivity, specificity, and accuracy were 0.88 (95% CI 0.72–1), 91.6% (62.0–100%), 85.7% (42.0–100%), and 89.0% (67.0–98.6%), respectively (Table 2).

**Table 2:** Performance of machine learning model and two experienced musculoskeletal (MSK) radiologists in distinguishing IM lipomas from atypical lipomatous tumors or well-differentiated liposarcomas

	AUC (95% CI)	Sensitivity (95% CI)	Specificity (95% CI)	Accuracy (95% CI)	*P* value*
Machine learning model	0.88 (0.72–1)	91.6 (62.0–100)	85.7 (42.0–100)	89.0 (67.0–98.6)	
MSK radiologist 1	0.94 (0.87–1)	97.4 (86.0–100)	90.9 (71.0–99.0)	95.0 (86.1–98.9)	0.4
MSK radiologist 2	0.91 (0.83–0.99)	100 (91.0–100)	81.8 (60.0–95.0)	93.3 (83.8–98.1)	0.7

*AUC* The area under the receiver operator characteristic curve, *CI* Confidence interval

*P value based on the Delong’s test, which were used to compare the AUCs between the machine learning model and radiologists

### Comparison between the machine learning model and two experienced MSK radiologists

**Fig. 5:**
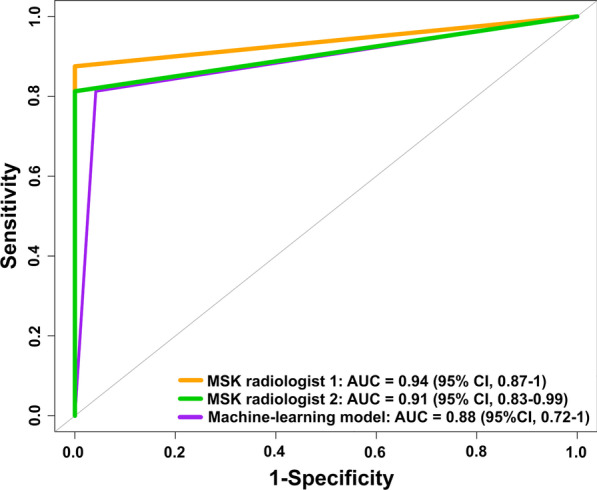
Comparison of performance between the machine learning model and experienced MSK radiologists in term of the area under the receiver operator characteristic curves (AUCs)

The classification performances of the 2 MSK radiologists are detailed in Table 2. The AUCs obtained by the radiologist 1 and 2 were 0.94 (0.87–1) and 0.91 (0.83–0.99). Their respective sensitivities, specificities, and accuracies were 97.4% (86.0–100%), 90.9% (71.0–99.0%), and 95.0% (86.1–98.9%), respectively, for radiologist 1 and 100% (91.0–100%), 81.8% (60.0–95.0%), and 93.3% (83.8–98.1%), respectively, for radiologist 2 (Table 2). Although the AUC of the machine learning model was lower than that from the two MSK radiologists, there was no statistically significant difference between the model and two radiologists (all *P* > 0.05) (Table 2 and Fig. 5).

### Fourteen feature importance insights in the ultimate model

**Fig. 6:**
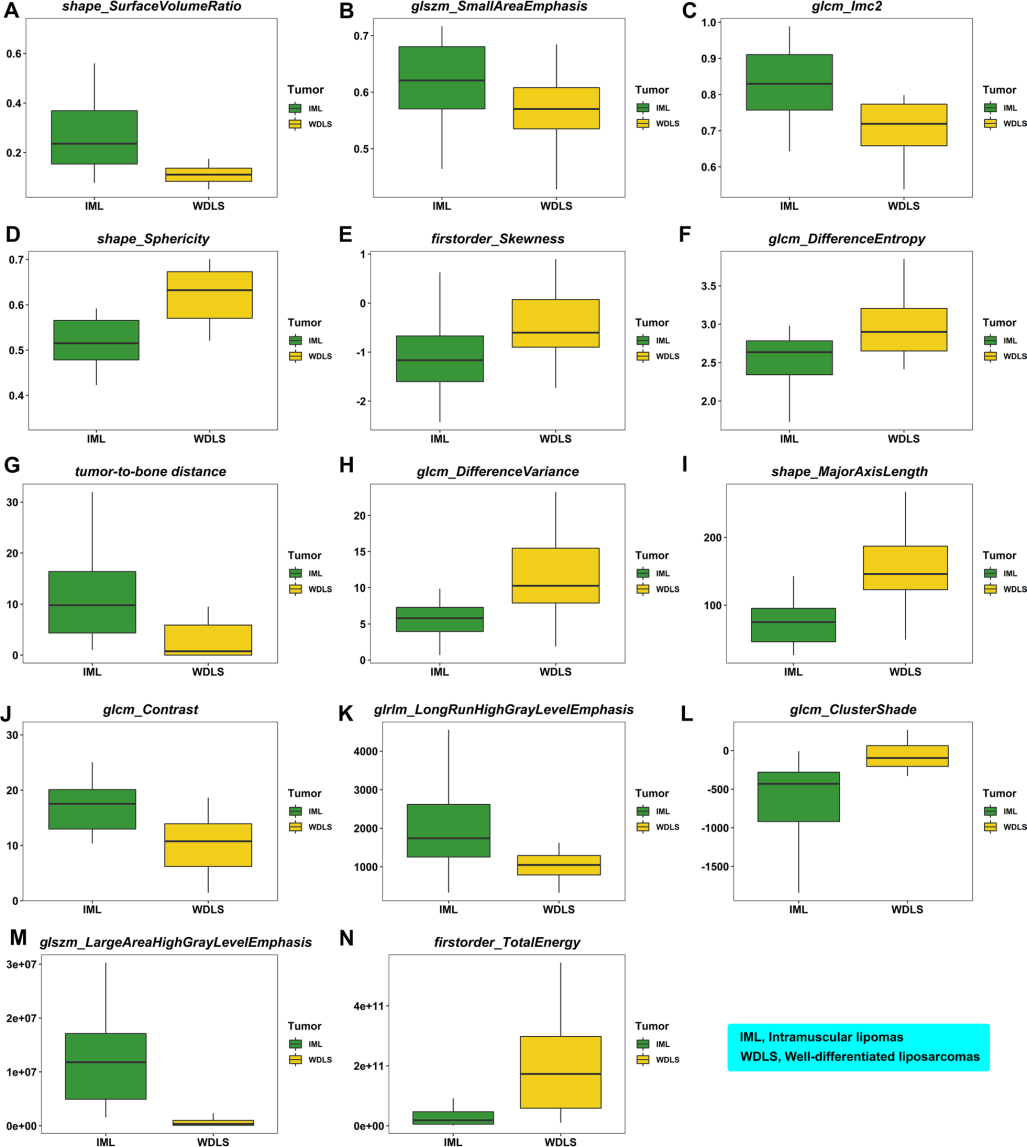
The boxplots of fourteen features (13 radiomic features and tumor-to-bone distance) with high importance that can identify soft tissue tumor differentiation: **A** shape_SurfaceVolumeRatio; **B** glszm_SmallAreaEmphasis; **C** glcm_Imc2; **D** shape_Sphericity; **E** firstorder_Skewness; **F** glcm_DifferenceEntropy; **G** tumor_to_bone_distance; **H** glcm_DifferenceVariance; **I** shape_MajorAxisLength; **J** glcm_Contrast; **K** glrlm_LongRunHighGrayLevelEmphasis; **L** glcm_ClusterShade; **M** glszm_LargeAreaHighGrayLevelEmphasis; **N** firstorder_TotalEnergy

Regarding analysis of feature importance, the 14 features were found to be significant after Bonferroni-corrected Mann–Whitney U test (Fig. 6, supporting information).

Three important shape-based features were (1) shape_SurfaceVolumeRatio (Fig. 6A), (2) shape_Sphericity (Fig. 6D), and (3) shape_MajorAxisLength (Fig. 6I), all of which suggested that ALTs/WDLS were more spherical and larger than IM lipomas.

Two histogram-based features of significance were (1) firstorder_Skewness (Fig. 6E) and (2) firstorder_TotalEnergy (Fig. 6N). The voxel intensity distribution showed negative skewness for IM lipomas and close to zero for ALTs/WDLSs. And the firstorder_TotalEnergy value for ALTs/WDLSs was higher than those for IM lipomas.

The 8 texture-based features indicated the degree of homogeneity and heterogeneity in the texture patterns in an image (Fig. 6B, C, F, H, J–M). These findings supported the position that IM lipomas exhibited more homogeneity in T1WI sequences than ALTs/WDLSs.

The tumor-to-bone distance (Fig. 6G) for IM lipomas was greater than those for ALTs/WDLSs.

## Discussion

This study revealed that regular MRI attainment facilitates the diagnosis of IM lipomas and WDLSs with credible results using the machine learning model based on tumor-to-bone distance and radiomic features derived from T1-weighted, non-contrast-enhanced MR images, with AUC 0.88 (95% CI 0.72–1), 91.6% sensitivity, 85.7% specificity, and 89% accuracy.

In this cohort of patients (Table 1), the mean ages of the patients with IM lipomas and ALTs/WDLSs were comparable. Women had IM lipomas (28 of 38 cases; 73.7%) more frequently than ALTs/WDLSs (14 of 30 cases; 46.7%), with statistically significance (*P* = 0.02). The most common location of the IM lipomas and ALTs/WDLSs was the thigh. Most calf tumors were ALTs/WDLSs, whereas tumors located in the shoulder, arm, back, trunk, and pelvis were almost always IM lipomas. Patient demographic data, including age, gender, and localization, may suggest malignancy. However, without a combination of clinical data, the machine learning approach based on tumor-to-bone distance and radiomic features of the T1W images themselves offer promising predictions of sarcoma.

Moreover, the present radiomics model required only T1W images without T2W or gadolinium contrast, which makes this method generalizable and reproducible for use in diverse centers equipped with various MRI scanners. No vendor effect was observed between the General Electric, Siemens, and Philips MR systems for any of the radiomic features in this study (Additional file 1: Table S1).

Three previous studies [17, 18, 21] (Table 3) evaluated all types of liposarcomas using a radiomic approach on MRI, including myxoid and dedifferentiated liposarcomas and a limited number of ALTs/WDLSs. Radiologists could easily differentiate the distinct radiological features of lipomas and high-grade liposarcomas on conventional MRI, resulting in high diagnostic accuracy (91–95%). In addition, Doyle et al. [13] showed that 2 radiologists achieved 94% and 100% sensitivities as well as 64% and 76% specificities for T1-weighted images when distinguishing between lipomas and atypical lipomatous tumor/WDLS. Focusing on studies of radiomics to differentiate lipomas from ALTs/WDLSs [19, 20, 22, 23], the accuracy dropped to 67%–96% because the lipomas and ALTs/WDLSs shared common MRI features. The results of our study are comparable with those of Vos et al. [22], who applied a radiomic model to differentiate both subcutaneous and IM lipomas from ATLs/WDLSs, using shape and texture analysis. Their design evaluated 58 lipomas and 58 WDLSs, with an accuracy of 67% when using T1W images and 75% when using combined T1W and T2W images. Recently, Tang et al. [20] reported that a radiomic models with T1WI, FS T2 WI, and T1&T2WI achieved 88%, 96%, and 92% accuracies when distinguishing between lipomas and ALTs of the extremities. Our non-contrast-enhanced T1W MRI achieved equivalent performance compared with those of Tang et al. [20], with an accuracy of 89%. This may be explained by using 3D segmentation to improve accuracy and usefulness while trading with a longer analysis time and GPU space [40].

**Table 3:** Comparing the performance of the classification in identifying soft tissue tumor differentiation in previous and current studies

Study	Number of patients with lipomas in benign group	Number of patients with ALTs/WDLSs in malignant group	Classify	Machine learning algorithm	AUC	Sensitivity (%)	Specificity (%)	Accuracy (%)
Cay, 2022 [15]	45	20/20 (100%)	Lipomas vs. ALTs/WDLSs	SVM	0.98	96.8	93.7	–
Fradet, 2022 [16]	40	45/45 (100%)	Lipomas vs. ALTs	Logistic regression	0.50	100	0.0	–
				SVM	0.47	70.0	32.0	–
				Random forest	0.71	64.0	68.0	–
				Gradient boosting	0.70	67.0	64.0	–
Tang, 2022 [20]	90	32/32 (100%)	Lipomas vs. ALTs	Random forest	0.94	85.7	100	96.0
Yang, 2022 [23]	69	58/58 (100%)	Lipomas vs. WDLSs	SVM	0.95	95.0	88.9	92.1
Malinauskaite, 2020 [18]	24	5/14 (35.7%)	Lipomas vs. Liposarcomas	SVM	0.93	88.8	100	94.7
				LDA	0.93	–	–	89.5
				Naïve Bayes	0.81	–	–	79.0
				Logistic regression	0.812	–	–	73.7
Leporq, 2020 [17]	40	?/41	Lipomas vs. Liposarcomas	SVM	0.96	100	90	95.0
Vos, 2019 [22]	58	58/80 (71.6%)	Lipomas vs. WDLSs	Various*	0.83	68	84	67.0
Thornhill, 2014 [21]	24	?/20	Lipomas vs. Liposarcomas	LDA	N/A	85	96	91.0
Current study								
Machine learning model	38	30/30 (100%)	IM lipomas vs. ALTs/WDLSs	LASSO LR	0.88	91.6	85.7	89.0
MSK radiologist 1	38	30/30 (100%)	IM lipomas vs. ALTs/WDLSs	-	0.94	97.4	90.9	95.0
MSK radiologist 2	38	30/30 (100%)	IM lipomas vs. ALTs/WDLSs	-	0.91	100	81.8	93.3

*IM lipomas* intramuscular lipomas, *ALTs/WDLSs* atypical lipomatous tumors/well-differentiated liposarcomas, *MSK radiologist* musculoskeletal radiologist, *LASSO LR* Least Absolute Shrinkage and Selection Operator (LASSO) logistic regression, *AUC* area under the curve, *N/A* not available

*The model was constructed using various methods including logistic regression, support vector machine (SVM), random forests, naïve Bayes, linear discriminant analysis (LDA), and quadratic discriminant analysis (QDA)

Our work compared the classification performances of the machine learning approaches and the radiologists. When looking at the prediction accuracy, the experienced MSK radiologists demonstrated superior performance to the machine learning model because of their great expertise. However, even though there was a small set of MRIs available for the learning process, the machine learning models required less time than the radiologists to perform their readings after the segmentation process was done. Additionally, when comparing the AUCs between the model and two radiologists in Fig. 5, it can be seen that there was no statistically significant difference. Therefore, the model based on tumor-to-bone distance and radiomic features has the potential to distinguish between IM lipomas and ALTs/WDLSs and its discrimination ability is close to the radiologists.

Regarding feature importance insights in the ultimate model, the 14 features importance indicated that shape-based, histogram-based, and texture-based features were of foremost importance (Figs. 4B and 6).

Shape-based features or size features were found to be related to the prediction of malignancy and were largely integrated into the models. The ALTs/WDLSs were generally larger than IM lipomas. This illustrated that tumor size may help to characterize malignancy, as reported by previous investigations based on conventional imaging characteristics [11, 12, 41, 42]. However, in some specific areas, such as an ALT/WDLS of the hand, the lesion is not large, and a large IM lipoma of the thigh is typically not difficult to find, depending on the location and looseness of the surrounding tissue. Therefore, radiographic size is not always a reliable radiomic feature for diagnosis. Although ovoid/nodular configurations cannot distinguish the grade of malignancy (i.e., high-, intermediate, and low-grade soft tissue tumors) on MRI [43], it may differentiate IM lipomas from ALTs/WDLSs. Based on our results, ALTs/WDLSs had a more compact (sphere-like) shape than IM lipomas, as evidenced by the surface area-to-volume ratio. This radiomic feature may be correlated to ovoid/nodular configurations on MRI [43, 44]. We can observe that this shape difference may also reflect excessive cell proliferation and/or non-lipomatous or nodular fibrous septa, which showed a fluffier consistency in ALTs/WDLSs [45, 46]. Tumor shape was also a key characteristic that was visually evaluated in clinical practice [12, 42]. Further studies with expanded data sets that include larger IM lipomas or smaller ALTs/WDLSs should be performed to identify important radiomic features in addition to the size- or volume-dependent features.

Regarding the histogram features, the higher heterogeneity in the skewness map of T1W values from tumor ROI correlated with a higher grade of malignancy. The ALTs/WDLSs had a greater value of energy feature than the IM lipomas.

Texture features (particularly the features of GLSZM, GLRLM, and GLCM) were largely reported herein as relevant features. This is also consistent with clinical practice based on visual characterization of tumor heterogeneity and the presence of thick and nodular septa as key characteristics [12, 47].

In addition to histogram and texture features, we found that depth played an important role. By measuring the tumor-to-bone distance on 3D reconstructions of T1W MR images from the closest aspect of the tumor to the bone (Fig. 2), we showed that the closer a tumor was to the bone, the higher the probability that it was an ALT/WDLS rather than an IM lipoma. This implies either that IM lipomas are often found in the more superficial muscle groups or that WDLS are larger, as mentioned earlier.

Because of the higher cost of FISH and its longer test turnaround time, molecular testing may be performed only in cases of clinical suspicion. Most ALT/WDLS cases were diagnosed by evidence of mature adipocytes with pleomorphism, nuclear atypia, and hyperchromatic stromal cells on hematoxylin and eosin stain. This method is widely accepted in our country. Using the machine learning approach may not replace molecular testing for MDM2 and CDK4. However, it may help to alert physicians to the need to refer a patient to a sarcoma center at the earliest opportunity. Thus, the use of the machine learning approach may also facilitate the development of plans for surgical treatment and follow-up.

We demonstrated that machine learning approach based on tumor-to-bone distance and radiomic features retrieved from 3D segmentation MRI can distinguish between IM lipomas and ALTs/WDLSs and provide quantitative information for their differential diagnosis. There are 3 main advantages of using the machine learning approach to differentiate between IM lipomas and ALTs/WDLSs before surgery. First, it obviates the need for a patient to undergo a biopsy. As mentioned above, biopsies of lipomatous tumors can produce unfavorable results because the foci of malignant cells that lie in the septum are difficult to reach. Another advantage is that this approach helps surgeons decide whether to wait or to accelerate the timing of surgery. This is because surgery on an IM lipoma can wait, but an ALT/WDLS cannot be left untreated for a long time as it has a 1–5% chance of becoming a dedifferentiated liposarcoma [5, 48, 49]. Furthermore, the use of the machine learning approach can guide the decision by a surgeon to use a minimally invasive incision (for an IM lipoma) or a larger incision with wider margins (for ALT/WDLS). Once an IM lipoma or ALT/WDLS has been resected and its diagnosis has been confirmed by histopathology, a different follow-up protocol is used for each. For an ALT/WDLS, a serial physical examination and/or MRI is performed every 3–4 months during the first 2 postoperative years and once a year for the following 3 years. This protocol is employed because WDLSs have a greater chance of recurrence and dedifferentiation to sarcomas than IM lipomas, but they rarely metastasize [6, 50, 51]. In contrast, IM lipomas require much less frequent follow-up. No additional treatment, including irradiation or chemotherapy, will be administered to either group.

Our study has some limitations. First, most of our IM lipomas were smaller in size. This volume bias was mentioned earlier in this article. Additionally, the sample sizes of 38 IM lipomas and 30 ALTs/WDLSs were relatively small, especially for diagnostic efficiency. We will continually evaluate the preoperative MRI examination of IM lipomas and ALTs/WDLSs to progressively refine our model. The model was based solely on selected retrospective data related to masses that had been surgically removed and for which pathological reports were available. However, small tumors treated with observation or masses that had not been referred to the sarcoma center were excluded from the data sets. Furthermore, we used manual 3D segmentation with semiautomatic contoured images by two observers (statistician and research scientist) which were confirmed by experienced MSK radiologists. This may be subject to intra- and interobserver variability; however, the results showed no significant difference between observers. Finally, we did not compare the different pathological subtypes of lipomas and ALTs/WDLSs (e.g., the sclerosing type). However, the subtype may not alter the surgical procedure.

## Conclusions

Quantitative analysis of an artificial intelligence-based system using machine learning approach based on tumor-to-bone distance and radiomic features derived from MRI images can differentiate between IM lipomas and ALTs/WDLSs. The predictive features that suggested malignancy were size, shape, depth, texture, histogram, and tumor-to-bone distance. This comprehensive study of multiple features contributes to the diagnosis of IM lipomas and ALTs/WDLSs. The further development of artificial intelligence-based systems that will be gained through training with a larger number of patients and improving the mathematical methods will improve the accuracy of these systems in diagnosing IM lipomas and ALTs/WDLSs.

## Supplementary Information

**Additional file 1**. **Table S1** Properties of the acquisition protocols of T1-weighted (T1W) MRI sequences of patients. **Table S2** Intra- and Interobserver variability of the radiomic features family and tumor-to-bone distance. **Table S3** Summary of histogram-based, shape-based, texture-based, and tumor-to-bone distance features with high importance that can distinguish between intramuscular (IM) lipomas and atypical lipomatous tumors/well-differentiated liposarcomas (ALTs/WDLSs).
