# Supplementary material for: Tumor-to-bone distance and radiomic features on MRI distinguish intramuscular lipomas from well-differentiated liposarcomas
Source: J Orthop Surg Res. 2023 Mar 28;18:255. doi: 10.1186/s13018-023-03718-4 (PMC10044811; doi:10.1186/s13018-023-03718-4)
Supplement: Supplementary file 1 — Additional file 1. Table S1 Properties of the acquisition protocols of T1-weighted (T1W) MRI sequences of patients. Table S2 Intra- and Interobserver variability of the radiomic features family and tumor-to-bone distance. Table S3 Summary of histogram-based, shape-based, texture-based, and tumor-to-bone distance features with high importance that can distinguish between intramuscular (IM) lipomas and atypical lipomatous tumors/well-differentiated liposarcomas (ALTs/WDLSs). [file 13018_2023_3718_MOESM1_ESM.docx]

**Appendix**

**Table S1** Properties of the acquisition protocols of T1-weighted (T1W) MRI sequences of patients.

| Property | Total  (n = 68) | IM lipomas  (n = 38) | ALTs/WDLSs  (n = 30) | *P* value* |
| --- | --- | --- | --- | --- |
| Magnetic field strength |  |  |  |  |
| 1.5T | 25 (36.8%) | 12 (31.6%) | 13 (43.3%) | 0.3 |
| 3.0T | 43 (63.2%) | 26 (68.4%) | 17 (56.7%) |  |
|  |  |  |  |  |
| Manufacturer |  |  |  |  |
| Philips | 51 (75.0%) | 29 (76.3%) | 22 (73.3%) | 0.8 |
| GE | 13 (19.1%) | 7 (18.4%) | 6 (20.0%) | 0.9 |
| Siemens | 4 (5.9%) | 2 (5.3%) | 2 (6.7%) | 0.9 |
|  |  |  |  |  |
| Setting (unit) |  |  |  |  |
| Slice thickness (mm) |  |  |  |  |
| Median (range) | 5 (3 - 8) | 5 (3 - 7) | 5 (3 - 8) | 0.2 |
|  |  |  |  |  |
| Repetition time (ms) |  |  |  |  |
| Median (range) | 590  (425 - 2,259) | 600  (425 - 1,425) | 569  (433 - 2,259) | 0.5 |
|  |  |  |  |  |
| Echo time (ms) |  |  |  |  |
| Median (range) | 12 (7 - 25) | 13 (7 - 25) | 12 (7 - 16) | 0.4 |
|  |  |  |  |  |
| Available the image that most clearly |  |  |  |  |
| Axial plane | 55 (80.9%) | 31 (81.6%) | 24 (80.0%) | 0.9 |
| Coronal plane | 11 (16.2%) | 6 (15.8%) | 5 (16.7%) | 0.9 |
| Sagittal plane | 2 (2.9%) | 1 (2.6%) | 1 (3.3%) | 0.9 |

IM lipomas, intramuscular lipomas; ALTs/WDLSs, atypical lipomatous tumors/well-differentiated liposarcomas.

**Table S2** Intra- and Interobserver variability of the radiomic features family and tumor-to-bone distance.

|  | Intraobserver variability | | | Interobserver variability | | |
| --- | --- | --- | --- | --- | --- | --- |
|  | ICC value  (with ICC > 0.75) | r | $\rho$ | ICC value  (with ICC > 0.75) | r | $\rho$ |
| Shape-based (13 features) | 0.95 ± 0.05 | 0.93 ± 0.07 | 0.94 ± 0.06 | 0.95 ± 0.06 | 0.92 ± 0.08 | 0.92 ± 0.08 |
|  |  |  |  |  |  |  |
| Histogram-based (18 features) | 0.90 ± 0.05 | 0.83 ± 0.09 | 0.84 ± 0.10 | 0.88 ± 0.05 | 0.83 ± 0.08 | 0.82 ± 0.11 |
|  |  |  |  |  |  |  |
| Texture-based |  |  |  |  |  |  |
| GLCM (23 features) | 0.91 ± 0.07 | 0.86 ± 0.08 | 0.88 ± 0.09 | 0.89 ± 0.06 | 0.84 ± 0.09 | 0.85 ± 0.09 |
| GLRLM (14 features) | 0.89 ± 0.07 | 0.84 ± 0.10 | 0.85 ± 0.10 | 0.89 ± 0.08 | 0.83 ± 0.13 | 0.82 ± 0.11 |
| GLSZM (14 features) | 0.89 ± 0.08 | 0.84 ± 0.11 | 0.86 ± 0.09 | 0.88 ± 0.08 | 0.85 ± 0.13 | 0.87 ± 0.11 |
| GLDM (1 feature) | 0.98 | 0.98 | 0.99 | 0.98 | 0.99 | 0.99 |
| NGTDM (1 feature) | 0.82 | 0.79 | 0.85 | 0.81 | 0.72 | 0.87 |
| Tumor-to-bone distance | 0.95 | 0.94 | 0.94 | 0.96 | 0.95 | 0.96 |

ICC, intraclass correlation coefficient

r, Pearson correlation coefficient

$\rho$, Spearman’s rank-order coefficient

The resulted values were presented using mean ± standard deviation

**Table S3** Summary of histogram-based, shape-based, texture-based, and tumor-to-bone distance features with high importance that can distinguish between intramuscular (IM) lipomas and atypical lipomatous tumors/well-differentiated liposarcomas (ALTs/WDLSs).

| Feature | Feature class name | Feature name | Formula | Definition |
| --- | --- | --- | --- | --- |
| Histogram-based | First order:    *When X is set of voxel intensity within a segmented ROI.* $X=\left\{x_{1},x_{2},x_{3},...,x_{N_{p}}\right\}$  | firstorder_Skewness | $skewness=\frac{\frac{1}{N_{p}}\sum_{i=1}^{N_{p}} (x_{i}-\bar{x})^{3}}{\left(\sqrt{\frac{1}{N_{p}}\sum_{i=1}^{N_{p}} (x_{i}-\bar{x})^{2}}\right)^{3}}$ | Measures the asymmetry of the distribution of voxel intensity within a segmented ROIs.  - Negative skewness indicates that the curve is extended towards the left side. (mean < median < mode)  - Skewness = 0, which means that the curve is a normal distribution.  - Positive skewness means that the curve is extended towards the right side. (mode < median < mean) |
|  |  | firstorder_TotalEnergy | $total\text{ }energy=V_{voxel}\sum_{i=1}^{N_{p}} \left(X_{i}+c\right)^{2}$,  where $c$is an optional value that is defined by voxelArrayShift), which shifts the intensities to prevent negative values in *X*. | Measures the value of energy feature that is scaled by the volume of the voxel in mm³.  In the case of a higher value, it presents that there is more the value of the energy feature. |
| Shape-based | Shape | shape_SurfaceVolumeRatio | *Surface area to volume ratio =* $\frac{A}{V}$,  where $A=\sum_{i=1}^{N_{f}} \left(\frac{1}{2}\left\vert a_{i}b_{i}\times a_{i}c_{i}\right\vert\right)$. $a_{i}b_{i}$  and $a_{i}c_{i}$are edges of the *i^th^* triangle in the mesh, formed by vertices $a_{i}$, $b_{i}$and $c_{i}$. *V* is shape_MeshVolume feature (i.e., the mesh volume in mm^3^ of the segmented ROI). $V=\sum_{i=1}^{N_{f}} \left(\frac{\left(Oa_{i}\left(Ob_{i}\times Oc_{i}\right)\right)}{6}\right)\text{ , }$  where $N_{f}$is the number of faces (triangles) defining the Mesh. For each face *i* in the mesh, defined by points $a_{i}$, $b_{i}$ and$c_{i}$, the (signed) volume $V_{f}$of the tetrahedron defined by that face and the origin of the image ($O$) is calculated. | In the case of a lower value, it indicates that there is a more compact (sphere-like) shape. |
|  |  | shape_Sphericity | . | Measures the roundness of the tumor shape relative to a sphere.  $0<sphericity\leq1$. $sphericity$  = 1, it indicates that the tumor shape is a perfect sphere. |
|  |  | shape_MajorAxisLength | $major\text{ }axis\text{ }length=4\sqrt{\lambda_{major}}$ ,  which is determined using the largest principal component $\lambda_{major}$ | This feature yield the largest axis length of ROI-enclosing ellipsoid. |
| Texture-based | Gray level co-occurrence matrix (GLCM):  *Where is the normalized co-occurrence matrix ($p\left(i,j\right)=\frac{P\left(i,j\right)}{\sum P\left(i,j\right)}$). $P\left(i,j\right)$ is the co-occurrence matrix for an arbitrary $\delta$ and $\theta$. $N_{g}$ is the number of discrete intensity levels in the image. $\varepsilon$is an arbitrarily small positive number ( $\approx2.2\times10^{-16}$). $p_{x_{i}}=\sum_{j=1}^{N_{g}} p\left(i,j\right)$ be the marginal row probabilities*  *$p_{y_{j}}=\sum_{i=1}^{N_{g}} p\left(i,j\right)$ be the marginal column probabilities.*  *$p_{x-y}\left(k\right)=\sum_{i=1}^{N_{g}} \sum_{j=1}^{N_{g}} p\left(i,j\right)$where $\left\vert i-j\right\vert=k$, and $k=0,1,2,...,N_{g}-1$ .$\mu_{x}$is the mean gray level intensity of $p_{x}$($\mu_{x}=\sum_{i=1}^{N_{g}} \left[\left(p_{x_{i}}\right)\left(i\right)\right]$). $\mu_{y}$is the mean gray level intensity of $p_{y}$($\mu_{y}=\sum_{j=1}^{N_{g}} \left[\left(p_{y_{j}}\right)\left(j\right)\right]$).* | glcm_Imc2 | Informational measure of correlation (IMC) 2 :  $\text{Imc2}=\sqrt{1-e^{-2\left(HXY2-HXY\right)}}$.  Where $HXY=-\sum_{i=1}^{N_{g}} \sum_{j=1}^{N_{g}} p\left(i,j\right)\log_{2}\left(p\left(i,j\right)+\varepsilon\right)$ be the entropy of $p\left(i,j\right)$.  $HXY_{2}=-\sum_{i=1}^{N_{g}} \sum_{j=1}^{N_{g}} p_{x_{i}}p_{y_{j}}\log{}_{2}p_{x_{i}}p_{y_{j}}+\varepsilon$. | Assesses the correlation between the probability distributions of *i* and *j* for quantifying the complexity of texture. $0\leq\text{Imc2 < 1}$     Imc2 = 0, which means that there is no mutual information. |
|  |  | glcm_DifferenceEntropy | Difference entropy = $\sum_{k=0}^{N_{g}-1} p_{x-y}\left(k\right)\log_{2}\left(p_{x-y}\left(k\right)+\varepsilon\right)$. | Measure the randomness/variability in neighborhood intensity value differences.  In the case of a larger value, it presents that there is more variability in neighborhood intensity value differences. |
|  |  | glcm_DifferenceVariance | Difference variance = $\sum_{k=0}^{N_{g}-1} \left(k-DA\right)^{2}p_{x-y}\left(k\right)$ | Measure the heterogeneity that places higher weights on differing intensity level pairs that deviate more from the mean. |
|  |  | glcm_Contrast | $contrast=\sum_{i=1}^{N_{g}} \sum_{j=1}^{N_{g}} \left(i-j\right)^{2}p\left(i,j\right)$ | Measure the local intensity variation, favoring values away from the diagonal (*i*=*j*).  In the case of a larger value, it presents that there is more correlation with a greater disparity in intensity values among neighboring voxels. |
|  |  | glcm_ClusterShade | Cluster shade = $\sum_{i=1}^{N_{g}} \sum_{j=1}^{N_{g}} \left(i+j-\mu_{x}-\mu_{y}\right)^{3}p\left(i,j\right)$ | Measure the skewness and uniformity of the GLCM.  In the case of a higher cluster shade value, it indicates that there is a greater asymmetry in the mean. |
|  | Gray-level run-length matrix (GLRLM):  *Where $N_{g}$is the number of discreet intensity values in the image $N_{r}$is the number of discreet run lengths in the image. $P(i,j)$ is the run-length matrix for an arbitrary direction* $\theta$*, when $i=1,2,3,...,N_{g}$and $j=1,2,3,...,N_{r}$.* | glrlm_LongRunHighGrayLevelEmphasis (*LRHGLE*) | $LRHGLE=\frac{\sum_{i=1}^{N_{g}} \sum_{j=1}^{N_{r}} P(i,j\vert\theta)i^{2}j^{2}}{N_{r}(\theta)}$ | Reflects graininess.  Measures the joint distribution of long run lengths with higher gray-level values.  In the case of a higher value, it indicates that there is more homogeneity with higher gray-level values. |
|  | Gray level size zone matrix (GLSZM):  *Where $N_{g}$is the number of discreet intensity values in the image. $N_{s}$is the number of discreet zone sizes in the image.$N_{p}$is the number of voxels in the image.$N_{z}$is the number of zones in the ROI, which is equal to $\sum_{i=1}^{N_{g}} \sum_{j=1}^{N_{s}} P(i,j)$ and $1\leq N_{z}\leq N_{p}$. $P(i,j)$ is the size zone matrix, when $i=1,2,3,...,N_{g}$and $j=1,2,3,...,N_{s}$.* | glszm_SmallAreaEmphasis (*SAE*) | $SAE=\frac{\sum_{i=1}^{N_{g}} \sum_{j=1}^{N_{s}} \left(\frac{P(i,j)}{j^{2}}\right)}{N_{z}}$ | Measure the distribution of small size zones.  In the case of a greater value, it indicates that there are smaller size zones and more fine textures. |
|  |  | glszm_LargeAreaHighGrayLevelEmphasis (*LAHGLE*) | $LAHGLE=\frac{\sum_{i=1}^{N_{g}} \sum_{j=1}^{N_{g}} P\left(i,j\right)i^{2}j^{2}}{N_{z}}$ | Measure the proportion in the image of the joint distribution of larger size zones with higher gray-level values.  In the case of a higher value, it indicates that there is more homogeneity in the texture patterns. |
| Tumor-to-bone distance | - | Tumor-to-bone distance | A Tumor-to-bone distance was measured in 3D (**Fig. 2**). | Tumor-to-bone distance in mm, distance from A to B is from the nearest aspect of the tumor (A) to the bone (B). |
